# Structural and functional characterization of a plant alpha‐actinin

**DOI:** 10.1002/2211-5463.13222

**Published:** 2021-06-25

**Authors:** Karina Persson, Lars Backman

**Affiliations:** ^1^ Department of Chemistry Umeå University Sweden

**Keywords:** actin‐binding protein, *Rhodamnia argentea*, spectrin repeat, α‐actinin

## Abstract

The Australian tree malletwood (*Rhodamnia argentea*) is unique. The genome of malletwood is the only known plant genome that contains a gene coding for an α‐actinin‐like protein. Several organisms predating the animal‐plant bifurcation express an α‐actinin or α‐actinin‐like protein. Therefore, it appears that plants in general, but not malletwood, have lost the α‐actinin or α‐actinin‐like gene during evolution. In order to characterize its structure and function, we synthesized the gene and expressed the recombinant *R. argentea* protein. The results clearly show that this protein has all properties of genuine α‐actinin. The N‐terminal actin‐binding domain (ABD), with two calponin homology motifs, is very similar to the ABD of any α‐actinin. The C‐terminal calmodulin‐like domain, as well as the intervening rod domain, are also similar to the corresponding regions in other α‐actinins. The *R. argentea* α‐actinin‐like protein dimerises in solution and thereby can cross‐link actin filaments. Based on these results, we believe the *R. argentea* protein represents a genuine α‐actinin, making *R. argentea* unique in the plant world.

## Graphical abstract

The Australian tree malletwood is so far the only known plant that expresses an alpha‐actinin or alpha‐actinin‐like protein. We show that the protein's N‐terminal actin‐binding domain (ABD) is functional and structurally very similar to other ABDs and that the C‐terminal calmodulin‐like domain contains two pairs of calcium‐insensitive EF‐hands. In the presence of filamentous, actin bundles or networks are formed.

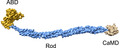

**Abbreviations**
ABD: actin‐binding domain
ABS: actin‐binding site
buffer TK: 50 mm Tris, pH 8.0, 200 mm KCl
CaMD: calmodulin‐like domain
Cb: carbenicillin
CH: calponin homology
FL: full length
TEV: tobacco etch virus

*Rhodamnia argentea* (malletwood) is a rain forest tree of eastern Australia. Like several other *Rhodamnia* species, *R. argentea* is endemic to Australia [1]. Recently, the *R. argentea* genome was completed, and highly unexpectedly, a gene coding for an α‐actinin‐like protein was found. Hitherto, a gene coding for an α‐actinin or an α‐actinin‐like protein has not been identified in any plant genome. Moreover, plants also apparently lack genes for the other members of the spectrin superfamily (such as spectrin and dystrophin) although cross‐reactivity of erythrocyte spectrin antibodies seems to imply the opposite [2, 3, 4, 5, 6, 7].

Alpha‐actinin is intimately related to contractile movement as it connects, directly or indirectly, the actin cytoskeleton to the Z disc in the muscle sarcomere and with membrane‐bound structures such as focal adhesion contacts. As α‐actinin forms antiparallel dimers, with an actin‐binding site (ABS) at each end of the dimer, it can also cross‐link actin filaments into bundles or networks that are important for the cellular infrastructure [8, 9, 10, 11, 12, 13, 14].

Mammals and fishes have four genes coding for distinct α‐actinins [15]. Two transcripts (ACTN2 and ACTN3) give rise to calcium‐insensitive α‐actinins and the other two (ACTN1 and ACTN4) code for calcium‐sensitive α‐actinins [9, 16, 17, 18]. Alternative splicing gives rise to additional transcripts, particularly of the calcium‐sensitive isoforms [19]. Birds have three α‐actinin genes, lacking ACTN3, whereas invertebrates and most fungi possess a single gene. The genome of *Saccharomyces cerevisiae* (Baker's yeast) as well as a few other fungi lack an α‐actinin gene. In Drosophila and probably most other invertebrates, alternative splicing of the single α‐actinin gene generates three isoforms with different tissue localization [20].

Independent of isoform, all analysed α‐actinins share the same domain structure, like the other members of the spectrin superfamily [8, 11, 18, 21, 22]. The domain structure contains an N‐terminal actin‐binding domain (ABD) composed of two calponin homology (CH1 and CH2) domains and a C‐terminal calmodulin‐like domain (CaMD) containing two lobes, each comprised of a pair of EF‐hands (EF1‐2 and EF 3‐4). A rod domain connects the N‐terminal ABD with the C‐terminal CaMD. Depending on the source of α‐actinin, the rod comprises four, two or a single spectrin repeat [15, 23]. In the antiparallel α‐actinin dimer, the C‐terminal CaMD of one monomer is placed close to the ABD of the other monomer. It has been suggested that calcium binding causes a conformational change in the CaMD, that affects the ABD affinity for actin binding [24, 25, 26].

Analysis of the *R. argentea* α‐actinin‐like gene sequence implies a protein with the common α‐actinin domain structure; a N‐terminal ABD, followed by four spectrin repeats and a C‐terminal CaMD. The amino acid sequence is more than 60% identical to the human isoforms and close to 70% identical to α‐actinin of *Drosophila*. To characterize this plausible α‐actinin from the plant world, we have cloned, expressed, and isolated the full‐length protein as well as the ABD and CaMD, in order to determine structure and functional properties.

## Materials and methods

### Cloning, expression and purification

The sequence of *R. argentea* α‐actinin‐like protein was obtained from NCBI, accession number XM_030655977, and used as template for gene synthesis. To facilitate subcloning, *BamHI* and *XhoI* restriction sites were added to the 5′‐ and 3′‐ends, respectively, of all synthesized genes. The full‐length gene as well as shorter fragments were synthesized and inserted in plasmid pEX‐a128 by Eurofins Genomics Germany GmbH (Ebersberg, Germany).

The resulting plasmids were used to transform competent *Escherichia coli* DH5α cells. Transformed cells were cultured for 15 h at 37 °C in LB medium containing 100 μm carbenicillin (LB/Cb). Plasmids were isolated using QIAprep Spin Miniprep Kit (Qiagen GmbH, Hilden, Germany).

Gene fragments were excised by *BamHI* and *XhoI*, gel purified, and ligated into pET‐TEV (containing an N‐terminal 10xHis‐tag and a TEV protease cleavage site) by the Protein Expertise Platform (Umeå University, Umeå, Sweden). The resulting plasmids, pTEV‐Rargentea‐ACTN, pTEV‐Rargentea‐ABD, and pTEV‐Rargentea‐EF, were then used to transform (by heat shock) competent *E. coli* BL21 (DE3). Due to the *BamHI* restriction site at the 5′‐end, two residues (Gly‐Ser) were added to all expressed proteins.

**Fig. 1:**
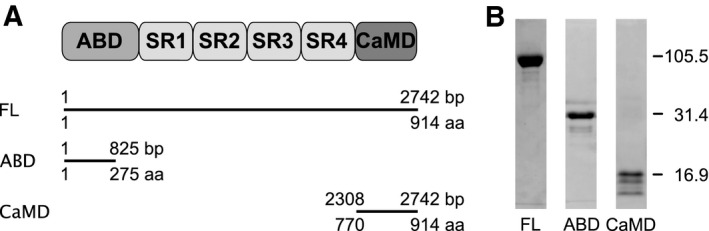
Domain organization of *Rhodamnia argentea* α‐actinin‐like protein. (A) The full‐length (FL) protein contains an N‐terminal ABD and a C‐terminal CaMD connected by a rod domain composed of four spectrin repeats (SR). (B) SDS/PAGE analysis of expressed and purified recombinant polypeptides.

The database Superfamily [27] and alignment with other α‐actinins were used to predict the structural domains of *R. argentea* α‐actinin‐like protein. The full‐length *R. argentea* α‐actinin‐like protein (ACTN) contains 914 residues. The ABD spans residues 1–275, and the calmodulin‐like C‐terminal domain (CaMD) spans residues 770–914, as shown in Fig. 1. Similar domain assignments were returned when Smart [28] and Pfam [29] were interrogated with the amino acid sequence, as Table 1 shows.

**Table 1:** Domain assignments of *Rhodamnia argentea* α‐actinin‐like protein by Superfamily, Smart and Pfam. The amino acid sequence of *R. argentea* α‐actinin was submitted to Superfamily [27], Smart [28] and Pfam [29].

	Superfamily		Smart		Pfam	
	Region	*E*‐value	Region	*E*‐value	Region	*E*‐value
Calponin homology domain	40–272	9.76e‐86	58–158	2.09e‐22	56–161	3.7e‐23
	–		171–270	2.01e‐24	169–276	1.3e‐25
Spectrin repeat 1	292–414	4.03e‐28	302–408	1.75	299–409	2.9e‐11
Spectrin repeat 2	415–524	3.44e‐24	422–523	9.82e‐22	419–524	1.3e‐22
Spectrin repeat 3	528–649	1.32e‐23	537–644	2.14e‐4	534–645	2.3e‐12
Spectrin repeat 4	651–764	2.71e‐20	658–757	0.154	655–758	4.1e‐10
EF‐hand	757–907	1.06e‐20	775–803	0.563	773–842	5.5e‐5
EF‐hand			816–844	0.0575		
Ca^2+^‐insensitive EF‐hand			847–911	9.91e‐30	847–911	3.4e‐21

For expression, transformed cells were cultured at 37 °C in LB/Cb medium until an optical density of 0.6–0.8 at 600 nm was reached. By addition of isopropyl thio‐β‐d‐galactoside to a final concentration of 0.5 mm protein expression was induced. After overnight culture at 16 °C, cells were harvested by centrifugation (29 000 ***g*** for 25 min), resuspended in 25 mm sodium phosphate buffer, pH 7.6, and 150 mm NaCl (NaPB‐NaCl). Resuspended cells were stored at −20 °C until purification.

To purify expressed protein, frozen cells were thawed and polyethylenimine was added to a final concentration of 0.05% before sonication on ice. Directly after sonication, 1/10th volume of 10% Triton X‐100 was added and the lysed cells were incubated on ice for ca 30 min. Cell debris was removed by centrifugation (58 000 ***g*** for 20 min), and imidazole was added to the clarified supernatant to give 10 mm final concentration. The protein solution was then loaded on a HiTrap™ Chelating FF column (GE Healthcare Bioscience AB, Uppsala, Sweden) charged with nickel. Unbound proteins were eluted with NaPB‐NaCl containing 100 mm imidazole, followed by an imidazole gradient ranging from 100 to 500 mm imidazole in NaPB‐NaCl to elute bound protein. Imidazole was removed by gel filtration on a HiPrep 26/10 desalting column (GE Healthcare Bioscience AB). When required, tobacco etch virus (TEV) protease (kindly provided by D. S. Waugh) was used to remove the His‐tag. Affinity chromatography on a nickel‐charged column was used to remove the released 10xHis‐tag and the 6xHis‐tagged TEV protease. Gel filtration on a HiPrep 26/10 desalting column was used to transfer the purified protein into buffer TK (50 mm Tris, pH 8.0, 200 mm KCl).

When required, further purification was achieved by gel filtration in buffer TK on Sephacryl 26/60 S‐100. Protein was routinely concentrated by Amicon Ultra centrifugal filter devices (MilliporeSigma, Burlington, MA, USA).

Protein concentration was determined by measurement of the absorbance at 280 nm using the molar attenuation coefficient (formerly molar absorptivity) as calculated from the amino acid sequence using ProtParm at the ExPASy Bioinformatics Resource Portal. Protein purity was routinely determined by denaturating SDS/PAGE [30].

### Actin co‐sedimentation assay

A co‐sedimentation assay was used to assess the actin‐binding and cross‐linking activity of *R. argentea* α‐actinin‐like protein. Actin was purified from acetone powder of rabbit skeletal muscle as described before [31, 32]. Actin, in 5 mm Tris/HCl, pH 8.0, 0.2 mm CaCl_2_, and 0.2 mm ATP, was allowed to polymerize at room temperature after addition of KCl and MgCl_2_ to final concentrations of 50 and 2 mm, respectively.

Cross‐linking activity was assayed by low‐speed co‐sedimentation. Under such conditions, only cross‐linked actin will sediment together with the cross‐linker. For this, first both polymerized (filamentous) actin and *R. argentea* α‐actinin‐like protein were centrifuged at 13 000 r.p.m. (16 000 ***g***) for 15 min before assay to remove aggregated protein. Filamentous actin was then mixed with *R. argentea* α‐actinin‐like protein and incubated at 20 °C for 60 min. After incubation, the mixture was centrifuged at 13 000 r.p.m. (16 000 ***g***) for 15 min protein and the supernatant and pellet were analysed by SDS/PAGE [33].

Actin‐binding activity was assayed by high‐speed co‐sedimentation. The rationale being that any protein that binds to filamentous actin will co‐sediment with actin. The expressed ABD of *R. argentea* α‐actinin‐like protein was centrifuged for 60 min at 90 000 r.p.m. (350 000 ***g***) to remove aggregated protein. The ABD was then mixed with filamentous actin and incubated at 20 °C for 60 min. The mixture was then centrifuged at 90 000 r.p.m. (350 000 ***g***) for 60 min, to sediment filamentous actin and actin‐bound proteins. The supernatant and pellets were analysed by SDS/PAGE.

### Negative staining transmission electron microscopy

Actin‐binding was also analysed by transmission electron microscopy. Leica EM ACE200 (Leica Microsystems, Wetzlar, Germany) carbon coating system was used to prepare copper grids coated with formvar. Grids were glow‐discharged with Pelco easiGlow system (Ted Pella, Inc., Redding, CA, USA). 3.5 μL sample was placed on the grid and absorbed for 2 min, washed twice in water, and stained immediately in 50 μL 1.5% uranyl acetate solution for 30 s. Samples were examined using FEI Talos L120 TEM equipped with a Ceta CMOS 4000 × 4000 pixels camera (Thermo Fisher Scientific, Hillsboro, OR, USA).

### CD spectroscopy

Expressed protein in buffer TK with or without 10 mm calcium was analysed by CD spectroscopy using a Jasco J‐810 spectrometer (Kovalent, Stockholm, Sweden). Spectra between 200 and 260 nm were collected using 0.025 nm step‐size and a scan speed of 50 nm·min^−1^, with a response time of 0.5 s and a bandwidth of 1 nm.

The temperature stability of expressed protein was determined by the ellipticity at 222 nm. The thermal scan rate was 1 °C·min^−1^, with a data pitch of 0.2 °C, 2 nm bandwidth, and 4 s response time.

### Isothermal calorimetry

Calcium affinity was also determined by isothermal calorimetry using a MicroCal iTC200 (Malvern Products, Uppsala, Sweden). The measuring cell was loaded with 0.13 mm *R. argentea* CaMD protein in 50 mm Tris/HCl, pH 8.0, 200 mm KCl, and the injection syringe was filled with 5–20 mm calcium chloride in the same buffer. Before measurement, the *R. argentea* CaMD protein solution was decalcified by passing over a Chelex 100 column.

Previous, it has been shown that the residual calcium levels in concentrated protein samples are very low (less than 0.02 mol calcium ions per mole of protein) even in sample untreated with Chelex 100 [34].

Injection profiles were analysed using nicpic [35] and sedphat [36].

### Crystallization and structure determination of the actin‐binding domain

Sitting‐drop vapour diffusion trials were performed at room temperature in MRC‐crystallization plates (Molecular Dimensions, Sheffield, UK). Droplets of 0.2 µL protein at 10 mg·mL^−1^ were mixed with 0.1 µL screening solution from Molecular Dimensions (Structure Screen I & II and MIDAS Plus) and Hampton Research (Aliso Viejo, CA, USA) (SaltRX). The final crystallization condition was 3.1 m sodium formate and 0.1 m sodium acetate pH 4.5.

Crystals were soaked in crystallization solution supplemented with 15% glycerol before they were flash cooled in liquid nitrogen and stored until data collection. X‐ray diffraction data were collected on an Eiger 16M detector (DECTRIS, Baden‐Dättwil, Switzerland) at beamline X06SA, Swiss Light Source. The diffraction images were processed with xds [37] and scaled with aimless from the ccp4 program suite [38]. The *R. argentea* ABD sequence was used to search the protein data bank for related proteins. The best hit (83% sequence identity) human actinin2 ABD, ACTN2 (pdb: 5a36) [39], was used as start model for molecular replacement using phaser [40]. The model was built and refined using iterative cycles of coot [41] and phenix.refine [42]. Hydrogen atoms were included and refined in the final model. Statistics for data collection, processing, and refinement are summarized in Table 2.

**Table 2:** Data processing and refinement statistics.

	Actin‐binding domain
Data collection	
Space group	P 3_1_ 2 1
Cell dimensions	
*a*, *b*, *c* (Å)	37.22, 37.22, 290.27
α, β, γ (°)	90.00, 90.00, 120.00
Resolution (Å)*	48.38–1.53 (1.53–1.50)
*R*_merge_	0.094 (0.862)
*I*/σ*I*	15.5 (2.9)
Total number of observations	728 797 (30 482)
Unique reflections	39 298 (1855)
Completeness (%)	99.9 (99.4)
Redundancy	18.5 (16.4)
CC1/2	0.999 (0.907)
Molecules in a.u.	1
Refinement	
Resolution (Å)	48–1.50
No. Reflections (work/test)	68 372/3578
*R*_work_/*R*_free_ (%)	16.7/19.0
No. atoms	
Protein	1951
Ligand/ion	23
Water	174
*B*‐factors (Å^2^)	
Protein	29.5
Ligand/ion	41.1
Water	36.9
R.m.s. deviations	
Bond lengths (Å)	0.010
Bond angles (°)	1.102
Ramachandran Favoured (%)	97.1
PDB code	7aw8

* = Highest resolution shell.

### Structure prediction

The amino acid sequence was submitted to several structure prediction web servers such as RaptorX [43], IntFOLD [44], Robetta [45], I‐Tasser [46], LOMETS [47], Phyre [48], and Quark [49] to predict the tertiary structure of separate domains.

ucsf chimera package was used to analyse structures and draw figures [50].

## Results and Discussion

### Cloning, expression and purification

The amino acid sequence of the *R. argentea* α‐actinin‐like protein was retrieved from NCBI. Based on the amino acid sequence of the ABD, CaMD and full‐length protein, corresponding DNA sequences were synthesized and subcloned into the expression vector (pET‐TEV).

The full‐length and ABD constructs expressed rather well whereas CaMD expressed poorly. All constructs could be purified to reasonable purity by immobilized metal affinity chromatography on nickel‐loaded columns. All purified proteins were soluble but degraded to varying degrees even in the presence of protease inhibitors. CaMD in particular exhibited fast degradation. Immediately after the affinity chromatography, three strong bands were observed on SDS/PAGE, as can be seen in Fig. 1. With time the largest band, representing the complete CaMD (16.9 kDa) disappeared and the other two bands increased in intensity. As these three peptides bound to the affinity column, degradation appears to occur at the C‐terminal part of CaMD, thus retaining the N‐terminal part of CaMD.

### Crystallization and structure determination

After initial crystallization screening and optimization, diffraction quality crystals were obtained. The crystals belonged to space group P3_1_21 with a single ABD per asymmetric unit. The structure was solved by molecular replacement followed by iterative manual model building and refinement to 1.5 Å resolution. The final *R*_work_/*R*_free_ was 16.7/19.0%. The model consists of residues 37–257, using numbering based on the full‐length *R. argentea* α‐actinin. Overall, the electron density was of high quality except for residues Val133‐Ser134. Data processing and refinement statistics are summarized in Table 2.

### Structure of the N‐terminal actin‐binding domain

**Fig. 2:**
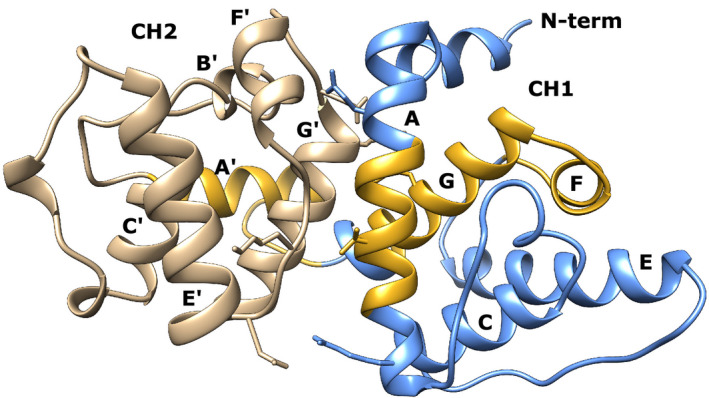
The structure of the ABD of *Rhodamnia argentea* α‐actinin‐like protein. The α‐helices of CH1 and CH2 are depicted in blue and tan, respectively. ABS1–3 are highlighted in gold.

Similar to previously published ABD structure, the *R. argentea* ABD is an all‐helical structure divided into two CH domains (CH1 and CH2). CH1 comprises helix A, C, E, F, and G and CH2 comprises helix A′, B′, C′, E′, F′, and G′. The two domains are linked by helices G and A′, whereas the main interaction surface is located on helix A and G′. Three actin‐binding sites are found in ABDs (ABS1–3), of which two are located in the CH1 domain and one in the linker and CH2 domain [51, 52]. ABS1 and ABS2 correspond to helix A and helices G and F of CH1, respectively, and ABS3 spans part of the linker between CH1 and CH2 and helix A′ of CH2 (Fig. 2). ABS1 is partially buried in the ‘closed’ conformation and becomes fully accessible after a rearrangement of the CH1–CH2 interface [52, 53, 54].

**Fig. 3:**
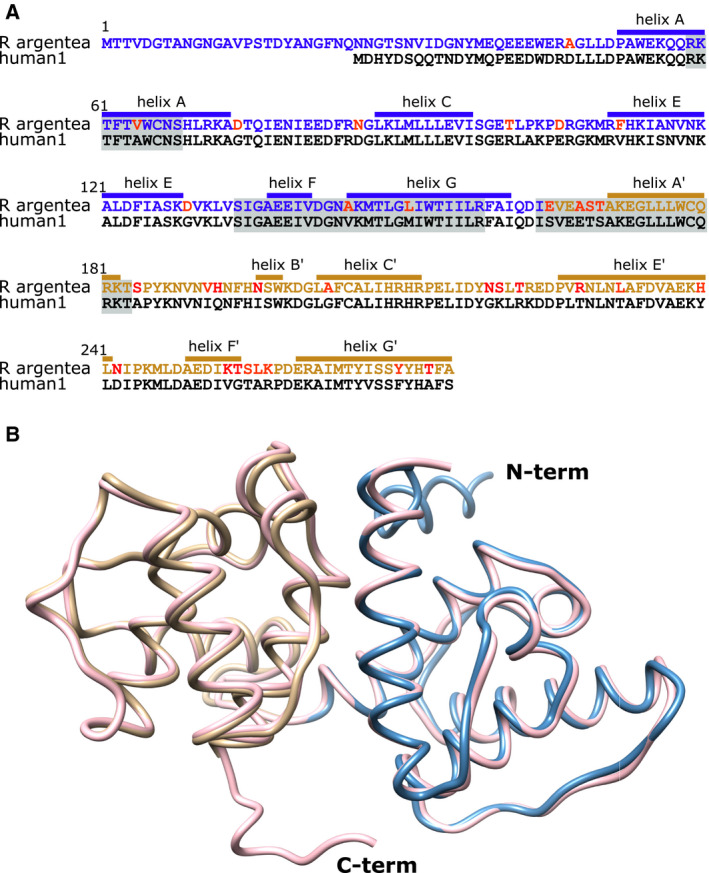
Comparison between *Rhodamnia argentea* α‐actinin‐like protein and human α‐actinin1 (A) Amino acid sequences of *R. argentea* α‐actinin‐like protein ABD aligned with the ABD of human α‐actinin1. The sequence of the two calponin homology domains (CH1 and CH2) is blue and tan, respectively. The helices are labelled as in Fig. 2. The three actin‐binding sequences (ABS1–3) are marked with a grey box and sequence differences in red. (B) The backbone of the ABD of *R. argentea* α‐actinin‐like protein (CH1: blue and CH2: tan) superimposed on the ABD of human α‐actinin1 (pink) (pdb: 2eyi).

By analysing its protein sequence it is clear that, except for the first 40 amino acid residues in the N‐terminus, the *R. argentea* ABD protein sequence is very similar to other α‐actinins' ABD sequences. In most cases, a polar or nonpolar residue in one ABD corresponds to a similar residue in the other ABD (Fig. 3A), keeping the same chemical characteristics. Therefore, it was not surprising that the crystal structure of *R. argentea* ABD overlapped nearly perfect with the structure of ABDs of other α‐actinins. When the structure of *R. argentea* ABD was submitted to the Dali server [55], the ABD of the four human α‐actinins was top scorers, with *Z*‐scores of more than 35 and root mean deviations of less than 1 Å. Figure 3B shows the backbone of *R. argentea* ABD superimposed on the ABD of human α‐actinin1.

Most of the sequence differences are outside the three ABSs [52, 53, 54]. Val64 in ABS1 and Ala145 and Leu151 in ABS2 of *R. argentea* α‐actinin‐like protein correspond to alanine and valine and methionine, respectively, in the ABD of human α‐actinin1 (Fig. 3A). In ABS3, the residues in human α‐actinin corresponding to Glu165 and Ala168 in *R. argentea* α‐actinin‐like protein are serine and glutamate, respectively. Thus, a hydroxyl‐containing side chain is changed for a charged and longer side chain and long charged side chain is changed for a small nonpolar one. If ABS3 is important for binding as has been proposed, these differences may affect the affinity.

The interface between CH1 and CH2 is in general conserved in most ABDs, so also in the ABD of *R. argentea* α‐actinin‐like protein. In the ABD of human α‐actinin1, Trp128 and Ile136 both interact with Met239 [52]. Further contacts between CH1 and CH2 involve hydrogen bonds between Gln32 and Asp234, Gln33 and Asp234, Thr36 and Met221, and Ala135 and His247 as well as a salt bridge between Arg46 and Asp217. All these interacting pairs are reproduced in the ABD of *R*. *argentea* α‐actinin‐like protein except for the salt bridge. Thus, Trp153 and Ile 161 interact with Met264, and Gln57, Gln58, Thr61, and Ala160 form hydrogen bonds with Asp259, Asp259, Met246, and His272. Instead of the aforementioned salt bridge, a hydrogen bond is present between Arg71 and Asn242. The hydrophobic (π‐cation) contact between Trp128 and Lys236 in human α‐actinin1 as well as in several other ABDs, that is proposed to function as a hinge between CH1 and CH2 [52], is preserved in *R. argentea* α‐actinin‐like protein although lysine is exchange for an arginine (Arg261). Therefore, it is likely that the *R. argentea* α‐actinin‐like protein undergoes a similar ‘opening’ of the closed form as other α‐actinins when binding to actin filaments [56].

### Binding actin filaments

By forming antiparallel dimers, with an ABD at each end, α‐actinin can cross‐link actin filaments into bundles or networks. It is well known that dimer formation requires the rod domain [57, 58]. The ability of *R. argentea* α‐actinin‐like protein to cross‐link actin filaments was examined using a low‐speed co‐sedimentation assay. In this assay, a mixture of actin filaments and the cross‐linker are centrifuged. A low‐speed centrifugation will pellet actin networks and bundles but not individual actin filaments. Thus, in the absence of cross‐linking activity, no actin will be pelleted and all actin end up in the supernatant after centrifugation. By analysing the distribution of actin and cross‐linker between supernatant and pellet, networking and bundling can be determined.

**Fig. 4:**
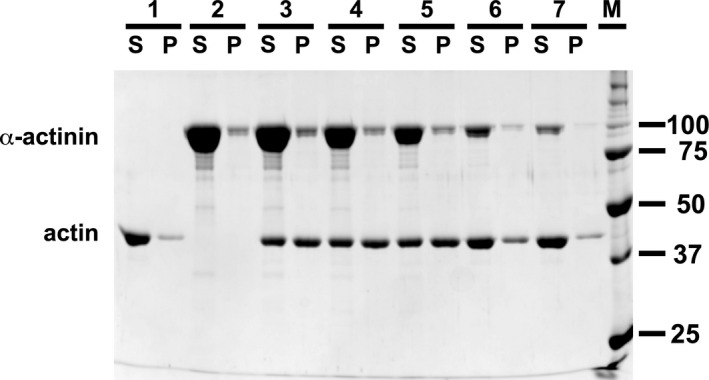
Actin binding. Filamentous rabbit skeletal muscle actin was incubated with varying concentrations of full‐length *Rhodamnia argentea* α‐actinin‐like protein for 60 min at room temperature and centrifuged for 15 min at 13 000 r.p.m (16 000 ***g***). Supernatants (S) and pelleted protein (P) were analysed by 10% SDS/PAGE. Lane 1: 5 μm actin; lane 2: 12.7 μm *R. argentea* full‐length α‐actinin‐like protein; lane 3: 5 μm actin and 12.7 μm α‐actinin‐like protein; lane 4: 5 μm actin 8.5 μm α‐actinin‐like protein; lane 5: 5 μm actin and 4.2 μm α‐actinin‐like protein; lane 6: 5 μm actin and 2.1 μm α‐actinin‐like protein; lane 7: 5 μm actin and 0.8 μm α‐actinin‐like protein; lane M: molecular size markers.

For this, mixtures of actin filaments and *R. argentea* α‐actinin‐like protein were incubated at room temperature before centrifugation at low‐speed (16 000 ***g*** for 15 min) followed by analysis of supernatant and pelleted material by SDS/PAGE. Figure 4 clearly shows that presence of *R. argentea* α‐actinin‐like protein causes actin and *R. argentea* α‐actinin‐like protein to be pelleted by a low‐speed centrifugation, whereas in its absence, actin is only present in the supernatant. Thus, the results imply that *R. argentea* α‐actinin‐like protein, like other α‐actinins, can cross‐link actin filaments.

**Fig. 5:**
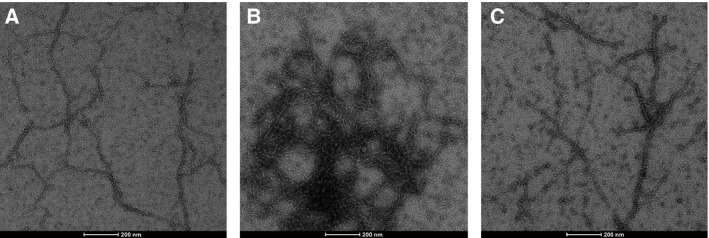
Negative staining transmission electron microscopy. 5 μm actin was incubated alone (A) or with 1.6 μm (B) and 0.5 μm (C) α‐actinin‐like protein before staining. Scale bar 200 nm.

This conclusion was collaborated by transmission electron microscopy. Figure 5 shows that *R. argentea* α‐actinin‐like protein crosslinks actin filaments into unordered networks (Fig. 5C). A higher ratio of cross‐linker causes formation of denser structures (or bundles) (Fig. 5B). In the absence of cross‐linker (Fig. 5A), the two‐start right‐handed helical structure of the actin filaments is clearly observable, whereas in the presence of *R. argentea* α‐actinin‐like protein, the two‐start helical structure is less clear. In the presence of the cross‐linker, many short (50–100 nm) structures are noticeable, that may represent actin filament fragments.

To determine whether the *R. argentea* ABD, like the ABD of any other α‐actinin, is required for binding to actin, a high‐speed co‐sedimentation assay was employed. In this case, a high‐speed centrifugation pellets actin filaments and any protein with affinity for actin will co‐sediment and will be detected in the pelleted material.

**Fig. 6:**
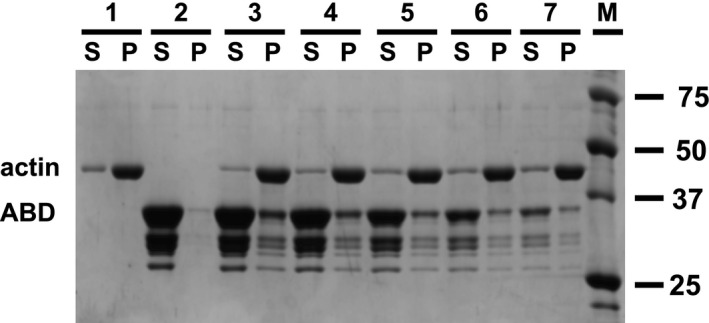
ABD binding to filamentous actin. Filamentous rabbit skeletal muscle actin was incubated with varying concentrations of the *Rhodamnia argentea* ABD for 60 min at room temperature followed by centrifugation for 60 min at 90 000 r.p.m (350 000 ***g***). Supernatants (S) and pelleted protein (P) were analysed by 12% SDS/PAGE. Lane 1: 5 μm actin; lane 2: 50 μm *R. argentea* ABD; lane 3: 5 μm actin and 50 μm ABD; lane 4: 5 μm actin and 37.5 μm ABD; lane 5: 5 μm actin and 25 μm ABD; lane 6: 5 μm actin and 12.5 μm ABD; lane 7: 5 μm actin and 6.3 μm ABD; lane M: molecular size markers.

To probe the affinity of the *R. argentea* ABD, mixtures of the ABD and filamentous actin were incubated at room temperature before centrifugation at high speed (350 000 ***g*** for 60 min). After centrifugation, supernatant and pellet were separated and analysed as before by SDS/PAGE. Figure 6 shows that in the presence of actin filaments a fraction of the *R. argentea* ABD is co‐sedimented together with actin. The results clearly indicate that the ability to bind and cross‐link actin filaments requires the ABD of *R. argentea* α‐actinin‐like protein.

### Calcium affinity of the C‐terminal calmodulin‐like domain

**Fig. 7:**
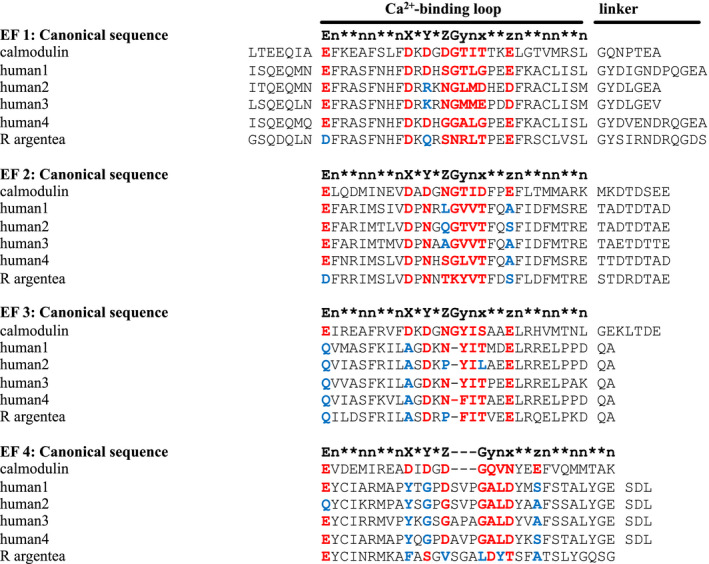
Calcium‐binding motifs. The sequence of the calmodulin‐like C‐terminal domain of *Rhodamnia argentea* α‐actinin‐like protein was aligned with C‐terminal domains of calmodulin and the four human isoforms as well as with the canonical sequence. In the binding loop, calcium is coordinated in a pentagonal bipyramidal configuration by residues at positions X, Y, Z, y, x and z. Residue X, Y, Z, x and z generally coordinate the calcium ion by side chain oxygens whereas residue y coordinates the ion through the backbone carbonyl oxygen. In the canonical sequence: E, glutamate; G, glycine; n, hydrophobic residue; *, any residue. Based on the Prosite PS00018 pattern: D‐x‐[DNS]‐{ILVFYW}‐[DENSTG]‐[DNQGHRK]‐{GP}‐[LIVMC]‐[DENQSTAGC]‐x(2)‐[DE], red indicates a residue present in active EF‐hand motifs, whereas blue indicates a residue not commonly found in this position of the calcium‐binding loop.

Both Pfam [29] and Smart [28] identify the N‐terminal EF‐hand pair as calcium‐active with weak *e*‐values and the C‐terminal EF‐hand pair as calcium‐insensitive with very low *e*‐values. Superfamily [27] recognizes the C‐terminal as an EF‐hand, with no indication whether it may be active or not (Table 1). When we aligned the *R. argentea* CaMD with those of known calcium‐binding EF‐hands, it was clear that important residues in all EF motifs required for calcium binding are missing. For instance, aspartate at position X and Y in the consensus sequence, present in all α‐actinins with a calcium‐sensitive EF motif, is missing in all *R. argentea* EF motifs, as Fig. 7 shows. Three of the EF motifs of *R*. *argentea* CaMD also lack the invariant glutamate in the entering α‐helix. From this, it is most likely that the *R. argentea* CaMD is unable to bind calcium ions.

**Fig. 8:**
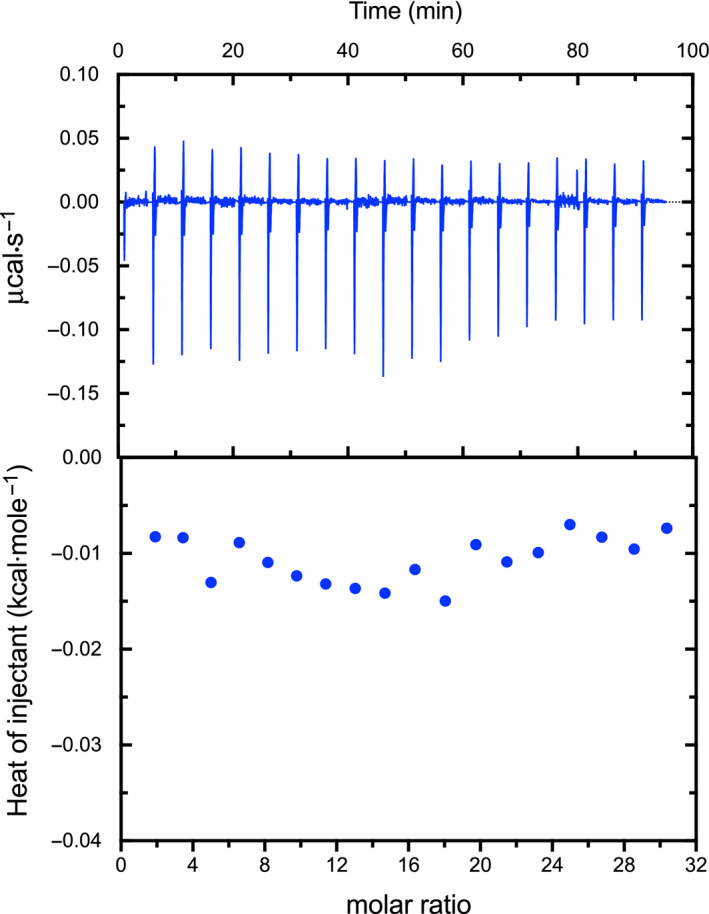
Isothermal calorimetry thermogram. Heat traces were obtained by injecting calcium chloride to *Rhodamnia argentea* CaMD. After integration, the obtained isotherm was flat and did not indicate a ligand binding event. The first point in the titration was not included in the analysis. Data shown are representative of three independent experiments.

This conclusion was supported by direct calcium‐binding analysis, both by isothermal calorimetry and folding stability. The heat traces obtained by injecting calcium to *R. argentea* CaMD did not show the typical profile of a ligand binding event, as evident in Fig. 8. Obtained traces were more or less indistinguishable from traces obtained by injecting buffer into buffer. The resulting ‘binding isotherms’ were flat and did not indicate any binding of the calcium ions, the CaMD.

**Fig. 9:**
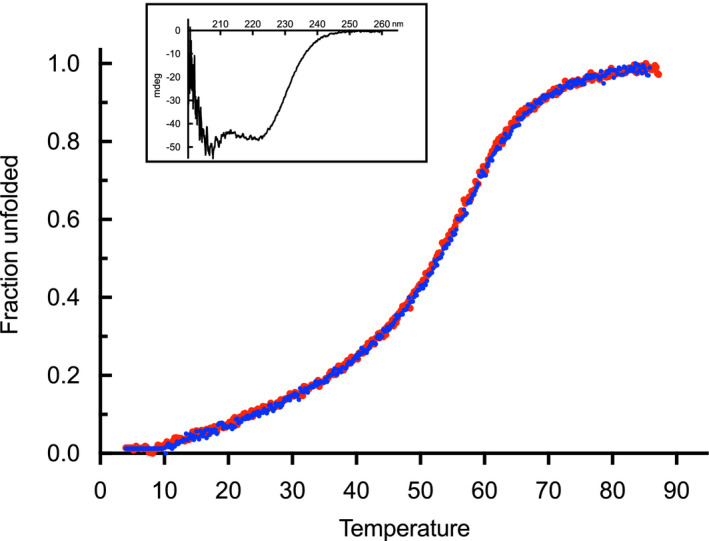
Thermal stability of the C‐terminal CaMD of *Rhodamnia argentea* α‐actinin‐like protein in the absence (red) and 10 mm calcium (blue) in 50 mm Tris – 200 mm KCl, pH 8 was determined from the molar ellipticity at 222 nm. Inset: Far UV CD spectrum of the C‐terminal CaMD of *R. argentea* α‐actinin‐like protein in 50 mm Tris – 200 mm KCl, pH 8.

As been shown before, binding of calcium increases the folding stability of the protein [59]. Therefore, temperature‐dependent unfolding was determined by CD in the absence and presence of calcium ions. The unfolding was clearly independent of the presence of calcium (Fig. 9). Thus, again indicating that the CaMD does not bind calcium ions.

It could be argued that the fast degradation of *R. argentea* CaMD may affect calcium binding. However, as degradation causes release of C‐terminal sequences, most likely the N‐terminal EF‐hands (EF1‐2 and probably EF3) are still intact even after degradation. Since EF1 is the active calcium‐binder in calcium‐sensitive α‐actinins, it seems very unlikely that the lack of calcium binding to *R. argentea* CaMD is due to degradation.

The amino acid sequence was submitted to several structure prediction web servers such as RaptorX [43], IntFOLD [44], Robetta [45], I‐Tasser [46], LOMETS [47], Phyre [48], and Quark [49]. Independent on the predictive algorithm, all services predicted similar structures with two globular lobes connected by a mainly α‐helical linker. However, the different services modelled the linker and loops in the lobes slightly differently. RaptorX and Robetta predicted the linker to be a continuous and nearly straight α‐helix, whereas the others predicted a helical linker with a nonhelical region in the middle.

**Fig. 10:**
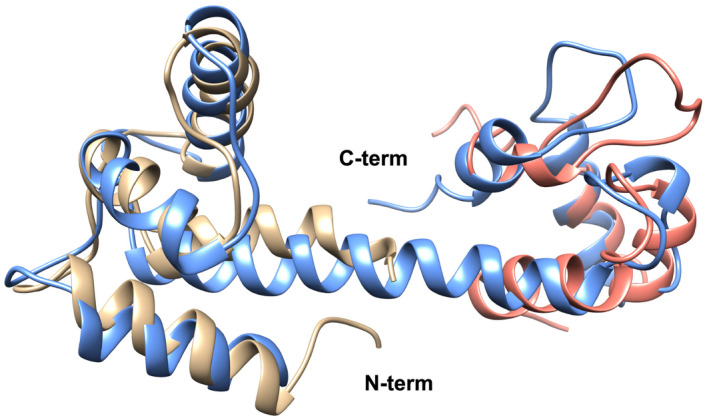
Predicted structure of the C‐terminal CaMD of *Rhodamnia argentea* α‐actinin‐like protein (blue) superimposed on the N‐terminal (tan) and C‐terminal (salmon) lobes of the C‐terminal CaMD of human α‐actinin1.

The model predicted by RaptorX was submitted to ReFOLD [60] for refinement (Fig. 10). The structure of the refined and final model was validated by the Protein Structure Validation Suite [61]. After refinement, according to both procheck [ 62] and molprobity [ 63], 97.7% of the residues were in favoured or allowed regions in the Ramachandran plot, whereas only four residues (Arg39, Asp41, Pro97, and Arg105) were in disallowed regions.

The overall structure of the predicted model is very similar to CaMDs of other α‐actinins, as expected due to the high sequence identity; the sequence identity with the same region of human α‐actinin1 is around 60% and the similarity is more than 86%. The N‐terminal lobe of the predicted CaMD superimposes on the N‐terminal half of human α‐actinin1 with a root mean deviation of less than 3 Å. The C‐terminal half is less similar structurally, with a root mean deviation close to 8 Å.

### Full‐length *R. argentea* α‐actinin‐like protein

**Fig. 11:**
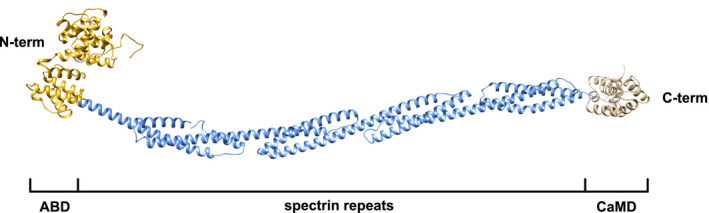
Model of full‐length *Rhodamnia argentea* α‐actinin‐like protein, with the N‐terminal ABD (golden), four spectrin repeats (blue), and the C‐terminal CaMD (tan).

To get a plausible view of the structure of the full‐length *R. argentea* α‐actinin‐like protein, we submitted the full‐length sequence to RaptorX. The predicted model is very similar to the determined α‐actinin structures of chicken gizzard muscle (pdb: 1sjj) and human muscle (pdb: 4d1e) α‐actinins, the hitherto only determined α‐actinin structures (Fig. 11).

The model includes the three typical α‐actinin domains; a N‐terminal ABD, a rod domain, containing four spectrin repeats, and a C‐terminal CaMD. Since the *R. argentea* α‐actinin‐like protein crosslinks actin filaments, it is highly likely that the native structure is composed of two molecules, forming an antiparallel homodimer, similar to all other characterized α‐actinins.

## Conclusion

It was indeed surprising to discover that the genome of *R. argentea* contains a transcribed gene that upon translation would give rise to an α‐actinin‐like protein. Our characterization of the *R. argentea* α‐actinin‐like protein has shown that the protein has all hallmarks of a typical α‐actinin: an N‐terminal ABD and a C‐terminal CaMD with an intervening rod domain with four spectrin repeats. The crystal structure of the ABD of *R. argentea* α‐actinin‐like protein superimposes very well on ABDs of α‐actinins. Also, the predicted model of the C‐terminal overlays well with the C‐terminal domain of α‐actinins. Although we have not characterized the rod domain in detail, based on sequence similarity, it is obvious that this part contains four triple‐helical repeats or so‐called spectrin repeats, similar to all animal α‐actinins.

Like any spectrin repeat, the spectrin repeats of *R. argentea* α‐actinin‐like protein contain about 110 residues. Also, the highly conserved tryptophan close to the N‐terminal (at position 17) of each repeat is present in *R. argentea* α‐actinin‐like protein. Therefore, we propose that the *R. argentea* α‐actinin‐like protein is in fact a genuine α‐actinin.

Since plants appear to lack α‐actinin, no published genome contains a sequence coding for α‐actinin or an α‐actinin‐like protein with the exception of *R. argentea* and *Quercus suber* (cork oak). However, the α‐actinin‐like protein of *Q. suber* (XM_024024281 and XM_024033408) contains a short rod domain, comprising of only two spectrin repeats, like α‐actinins of fungi. Since the α‐actinins of all higher eukaryotes have a rod domain comprising four spectrin repeats, it is possible that the annotated sequence is not from *Q. sube*r but rather a contaminating organism, possible of fungal origin.

*Entamoeba histolytica* (Entamoebidae), *Trichomonas vaginalis* (Parabasalid), *Phytophthora infestans* (Stramenopiles), and *Bigelowiella natans* (Chlorarachniophyte) as well as several other organisms that predates the animal‐plant bifurcation express an α‐actinin or an α‐actinin‐like protein [15, 59, 64, 65, 66]. Thus, plants, with the exception of *R. argentea*, appear to have lost the α‐actinin gene during the evolution. Whether other *Rhodamnia* species also have kept an α‐actinin gene is unknown due to the lack of sequence information.

It may be speculated that the presence of a *R. argentea* α‐actinin has given an evolutionary advantage. If so, it seems even more inexplicable why other plants have lost it.

## Conflict of interest

The authors declare no conflict of interest.

## Author contributions

LB and KP conceived and designed the project, acquired the data, analysed and interpreted the data and wrote the paper.

## Data Availability

The crystal structure of *R. argentea* α‐actinin ABD has been deposited in the Protein data bank with pdb code: 7aw8.
